# Crim1 regulates integrin signaling in murine lens development

**DOI:** 10.1242/dev.125591

**Published:** 2016-01-15

**Authors:** Ying Zhang, Jieqing Fan, Joshua W. K. Ho, Tommy Hu, Stephen C. Kneeland, Xueping Fan, Qiongchao Xi, Michael A. Sellarole, Wilhelmine N. de Vries, Weining Lu, Salil A. Lachke, Richard A. Lang, Simon W. M. John, Richard L. Maas

**Affiliations:** 1 Division of Genetics, Department of Medicine, Brigham and Women's Hospital and Harvard Medical School, Boston, MA 02115, USA; 2 Department of Developmental Biology, Cincinnati Children's Hospital Medical Center, Cincinnati, OH 45229, USA; 3 Center for Biomedical Informatics, Harvard Medical School, Boston, MA 02115, USA; 4 Victor Chang Cardiac Research Institute, and The University of New South Wales, Sydney, New South Wales 2010, Australia; 5 Howard Hughes Medical Institute and The Jackson Laboratory, 600 Main Street, Bar Harbor, ME 04609, USA; 6 Renal Section, Department of Medicine, Boston University Medical Center, Boston, MA 02118, USA; 7 Department of Biological Sciences, University of Delaware, Newark, DE 19716, USA

**Keywords:** Cataract, Cell adhesion, Cysteine-rich transmembrane BMP regulator 1, Crim1, Eye development, Integrin, Lens, Mouse

## Abstract

The developing lens is a powerful system for investigating the molecular basis of inductive tissue interactions and for studying cataract, the leading cause of blindness. The formation of tightly controlled cell-cell adhesions and cell-matrix junctions between lens epithelial (LE) cells, between lens fiber (LF) cells, and between these two cell populations enables the vertebrate lens to adopt a highly ordered structure and acquire optical transparency. Adhesion molecules are thought to maintain this ordered structure, but little is known about their identity or interactions. Cysteine-rich motor neuron 1 (Crim1), a type I transmembrane protein, is strongly expressed in the developing lens and its mutation causes ocular disease in both mice and humans. How Crim1 regulates lens morphogenesis is not understood. We identified a novel ENU-induced hypomorphic allele of *Crim1*, *Crim1*^glcr11^, which in the homozygous state causes cataract and microphthalmia. Using this and two other mutant alleles, *Crim1*^null^ and *Crim1*^cko^, we show that the lens defects in *Crim1* mouse mutants originate from defective LE cell polarity, proliferation and cell adhesion. Crim1 adhesive function is likely to be required for interactions both between LE cells and between LE and LF cells. We show that Crim1 acts in LE cells, where it colocalizes with and regulates the levels of active β1 integrin and of phosphorylated FAK and ERK. The RGD and transmembrane motifs of Crim1 are required for regulating FAK phosphorylation. These results identify an important function for Crim1 in the regulation of integrin- and FAK-mediated LE cell adhesion during lens development.

## Abstract (precis)

**Summary:** Crim1, a type I transmembrane protein, acts in lens epithelial cells where it colocalizes with and regulates the levels of active β1 integrin to control cell adhesion during mouse lens morphogenesis.

## INTRODUCTION

The developing lens is a powerful system for the study of tissue interactions and also the target of the medically important ocular disease cataract, a lens opacity that affects over 25 million individuals and is the leading cause of blindness worldwide (Asbell et al., 2005; Hejtmancik, 2008). The mature lens consists of two polarized cell types: a monolayer of lens epithelial (LE) cells and a mass of elongated and aligned lens fiber (LF) cells. The entire structure is covered by a lens capsule, a thick basement membrane secreted by LE and early LF cells in a polarized manner (Wederell and de longh, 2006). During development, the lens originates from a thickening of the head ectoderm that invaginates to form the lens pit, and then detaches to form the lens vesicle. Cells from the anterior lens vesicle differentiate into epithelial cells, while cells from the posterior lens vesicle elongate to form primary fiber cells. In later embryogenesis, LE cells continuously proliferate and differentiate into secondary fiber cells at the lens equator (Lovicu and McAvoy, 2005; McAvoy et al., 1999). Many cellular processes, including cell adhesion, actin dynamics, proliferation, differentiation and migration are important for lens transparency. The study of cell adhesion molecules reveals that contacts between LE and LE cells, LE cells and matrix, and between LE and LF cells are crucial for lens survival and for the maintenance of the LE cell phenotype (Pontoriero et al., 2009; Wederell and de longh, 2006). However, details of the molecular mechanisms involved are not well understood.

Members of the integrin family are implicated in the cell adhesion processes that occur in the developing lens. Integrins are the major cell adhesion transmembrane proteins that connect cells to the extracellular matrix (ECM) (Hynes, 1992). In mouse, there are 18 α and 8 β subunits that can form 24 different integrin heterodimers, each capable of preferentially binding a set of ECM substrates. Upon binding, integrins activate signaling pathways to transduce signals from outside the cell to inside, or vice versa, to regulate many cellular processes, including cell adhesion, proliferation, migration and differentiation. β1 integrin forms the largest integrin subfamily as it can assemble into heterodimers with 12 different α subunits. Studies of lens development have shown that β1 integrin is expressed in LE cells and LF cells (Bassnett et al., 1999; Menko and Philip, 1995; Wederell et al., 2005), whereas β3 and β4 integrins are also expressed in developing lens, together with αv and α6, respectively [reviewed by Walker and Menko (2009)]. Although knockout of the mouse β1 integrin gene (*Itgb1*) leads to peri-implantation lethality (Fassler and Meyer, 1995; Stephens et al., 1995), conditional knockout of *Itgb1* in lens results in cataract and microphthalmia due to apoptosis of LE cells and loss of the LE cell phenotype (Samuelsson et al., 2007; Simirskii et al., 2007). Immunofluorescence analysis of the *Itgb1* null lens shows that the epithelium becomes disorganized and begins to express the mesenchyme marker α-smooth muscle actin (Simirskii et al., 2007). Thus, integrin signaling can affect adhesion, actin dynamics and proliferation processes known to be important for lens morphogenesis, but understanding how other molecules integrate with or regulate integrin signaling in lens development remains incomplete.

Genetic mouse mutants can provide significant new and unbiased insight into the molecular mechanisms of lens development. From a forward N-ethyl-N-nitrosourea (ENU) mutagenesis screen, we scored novel mouse cataract phenotypes and identified a mutation that creates a cryptic splice acceptor within an intron to produce a hypomorphic allele of *Crim1*, *Crim1*^glcr11^. Crim1 is a type I transmembrane protein, with an N-terminal insulin-like growth factor-binding protein motif (IGFBP) and six cysteine-rich von Willebrand factor C (vWC) repeats located in the extracellular domain (Kolle et al., 2000). The six vWC repeats of Crim1 resemble those of extracellular proteins such as collagens VI, VII, XII and XIV, and of chordin, a BMP antagonist (Colombatti et al., 1993). *Crim1* mRNA is spatially and temporally regulated in various tissues and cell types, including the neural tube (Kolle et al., 2000), vascular system (Fan et al., 2014; Glienke et al., 2002), urogenital tract (Georgas et al., 2000), ear and eye (Lovicu et al., 2000; Pennisi et al., 2007). Mouse *Crim1* mutants display perinatal lethality with defects in limbs, kidney, vascular system and eye, and analysis of a *Crim1* null mutant suggests a role in maintaining retinal vascular and renal microvascular stability through Vegfa signaling (Fan et al., 2014; Wilkinson et al., 2007, 2009). Studies in *Xenopus* embryos show that the cytoplasmic domain of Crim1 can complex with N-cadherin and β-catenin and regulate adhesion complex stability in neural ectoderm (Ponferrada et al., 2012). Biochemical analysis of Crim1 has shown that it can act as a BMP antagonist by binding with BMPs and so inhibit their maturation and secretion (Wilkinson et al., 2003). Crim1 localizes to different subcellular compartments, including the endoplasmic reticulum, membrane compartments upon stimulation, and the secretory compartment (Glienke et al., 2002). The distinct localization of Crim1 and its unique structural motifs suggest that Crim1 executes multiple roles in development.

Recently, *CRIM1* haploinsufficiency was implicated in the human ocular syndrome MACOM (OMIM #602499), which is characterized by iris coloboma, microcornea, and increased axial length associated with myopia (Beleggia et al., 2015). Here we show that mice homozygous for any one of three *Crim1* loss-of-function mutations also exhibit striking defects in lens and ocular development. Using these three alleles, we demonstrate that Crim1 is required during lens development for the acquisition of LE cell polarity, for LE cell proliferation, and for appropriate cell-cell adhesive interactions required for organized lens development. We further show that Crim1 can bind to β1 integrin and that it regulates integrin, FAK and ERK signaling both in mouse lens tissue and in cultured cells. These results identify a novel role for Crim1 in the regulation of integrin and integrin-related downstream signaling during lens morphogenesis.

## RESULTS

### Identification of an intronic mutation in the *Crim1*^glcr11^ mouse mutant

**Fig. 1.**
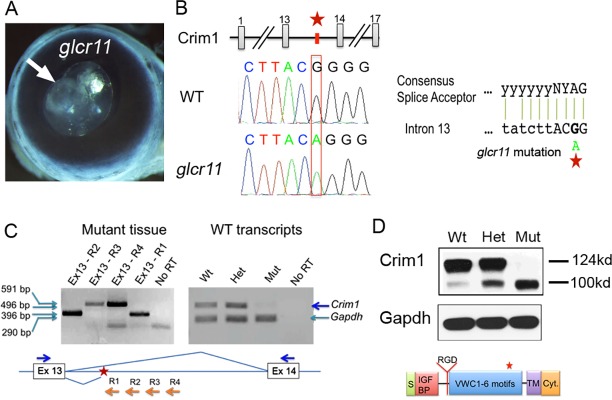
**WGS identifies an intronic mutation in *Crim1*^glcr11^ mouse cataract mutants.** (A) *glcr11* mutant mice exhibit cataract (arrow). (B) Sequencing shows a G→A mutation in *Crim1* intron 13, which creates a perfect cryptic consensus splice acceptor by creating a required A at the −2 position. (C) RT-PCR shows that the cryptically spliced transcripts continue at least 370 bp downstream of the mutation, truncating the exon 13 open reading frame and appending a short nonsense peptide. Upper blue line, normal splicing pattern; lower blue line, aberrant splicing pattern in the *Crim1*^glcr11^ mutant. R1-R4, reverse primers used for RT-PCR; RT, reverse transcriptase. (D) Western blot shows the full-length Crim1^glcr11^ protein at ∼124 kDa and a small amount of known proteolytic product (Wilkinson et al., 2003) at ∼100 kDa in the wild-type lens. In the *Crim1*^glcr11^ mutant, Crim1 is truncated via a stop codon shortly after exon 13, and hence the full-length 124 kDa form is absent. The truncated Crim1^glcr11^ protein is almost the same size (∼100 kDa) as the naturally occurring proteolytic form. Beneath is shown the Crim1 protein domains; the red asterisk indicates the truncation position in the *Crim1*^glcr11^ mutant. Panels A-D are representative of six independent experiments. IGFBP, insulin-like growth factor-binding protein motif; vWC, von Willebrand factor C repeats; TM, transmembrane domain; Cyt., cytoplasmic domain; RGD, Arg-Gly-Asp motif; S, signal peptide.

In a forward ENU screen we identified a recessive mouse mutant that exhibited cataract (Fig. 1A, arrow). This mutant, designated *glcr11* (*glaucoma relevant 11*) was mapped to an 8 Mb region on mouse chromosome 17 using strain-specific polymorphisms and meiotic recombination mapping (Fig. S1A). Whole-genome sequencing (WGS) identified eight homozygous variants within a larger 26 Mb region (Fig. S1A), with four variants in deep intergenic regions and four in introns. Of the genes that contained intronic variants, *Crim1* had the highest embryonic lens-specific expression according to the iSyTE gene expression database (Lachke et al., 2012). Furthermore, the *Crim1* variant, a homozygous G→A transition in intron 13, created a consensus splice acceptor motif (Dogan et al., 2007) that could constitute a cryptic splice acceptor (Fig. 1B). RT-PCR followed by DNA sequence analysis confirmed that this variant creates a functional cryptic splice acceptor site within intron 13 that truncates the *Crim1* open reading frame shortly after exon 13 via a stop codon in intron 13 and appends a short nonsense peptide (Fig. 1B,C). This variant is therefore a functional mutation, as verified by the size of the truncated Crim1 protein detected in *Crim1*^glcr11^ mice by western blot (Fig. 1D). To formally prove that the *Crim1* mutation is responsible for the *glcr11* phenotype, we obtained an existing *Crim1* null allele, *Crim1*^null^, and performed a classical complementation test with *Crim1*^glcr11^. The two alleles failed to complement, and *Crim1*^glcr11/null^ transheterozygotes displayed cataracts and other lens defects similar to those seen in homozygotes for each of the two alleles (Fig. S1B). Thus, *Crim1* loss-of-function causes the lens phenotype in *Crim1*^glcr11^ mutants.

**Fig. 2.**
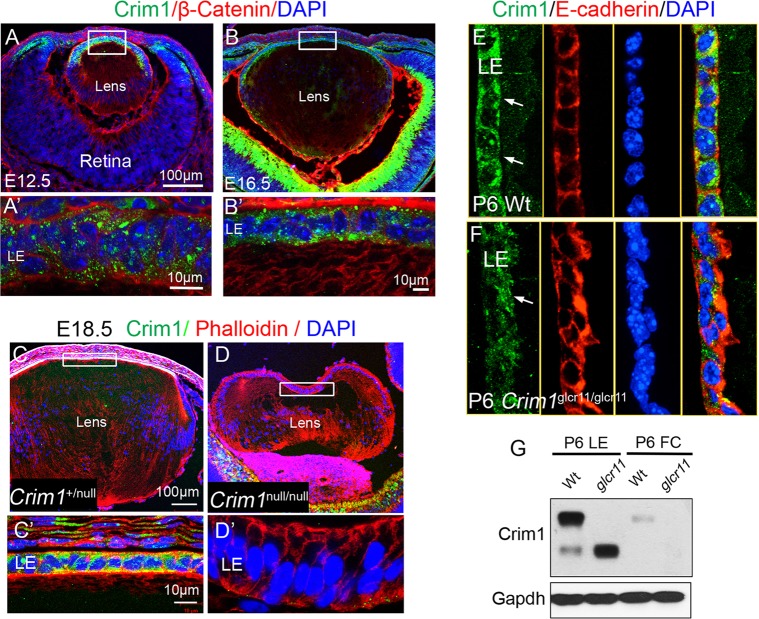
**Crim1 is localized to the cell membrane and cytoplasm of LE cells.** (A-B′) Crim1 is expressed in LE cells during lens development. High magnification of the boxed regions (A′,B′) shows that Crim1 localizes to the membrane and cytoplasm of LE cells. (C-D′) Validation of Crim1 antibody in *Crim1*^null^ lenses. High magnification shows that no Crim1 expression is detected in the *Crim1*^null^ LE cells (D′). (E,F) In P6 lenses, Crim1 expression becomes more concentrated in LE cell membranes and in cell-cell adhesions (E, arrows). In *Crim1*^glcr11^ mutants, Crim1 loses its membrane localization (F, arrow) and LE cell morphology is altered as indicated by the epithelial membrane marker E-cadherin (F, red panel). (G) Western blot showing that Crim1 is mainly detected in LE cells. Images shown are representative of four independent experiments.

We next examined Crim1 protein expression during mouse lens development. Crim1 expression is developmentally regulated, with expression beginning at E10.5 throughout the entire lens and then becoming concentrated in LE cells at E12.5 (Fig. 2A-E). By comparison, Crim1 expression in LF cells is minimal, as revealed by immunofluorescence (IF) and by western blot analysis (Fig. 2A-G), suggesting that Crim1 plays a crucial role in LE cells. When we stained *Crim1*^null^ lenses using anti-Crim1 antibody, no IF signal was detected in LE cells (Fig. 2D′). In wild-type mice, Crim1 localizes to the cell membranes and intracellular membranes of LE cells. By contrast, although residual intracellular expression persists, the membrane localization of Crim1 in *Crim1*^glcr11^ mutant LE cells is severely reduced, and staining for the epithelial adhesion junction marker E-cadherin showed disorganized LE cell-cell adhesions (Fig. 2F, arrow). The reduction in Crim1 levels in LE cells was confirmed by western blot, which showed a marked reduction in the full-length 124 kDa Crim1. Notably, Crim1 undergoes proteolytic cleavage just proximal to the transmembrane domain to generate an extracellular 100 kDa isoform (Wilkinson et al., 2003). The *Crim1*^glcr11^ splicing mutation occurs between the vWC5 and vWC6 domains and truncates Crim1 prior to its transmembrane and cytoplasmic domains. This markedly reduces the level of the 124 kDa isoform and generates a truncated mutant extracellular-domain-only form of Crim1 that migrates close in size to the naturally occurring 100 kDa Crim1 isoform generated by proteolytic cleavage (Fig. 2G).

### *Crim1*^glcr11^ mutants develop cataract and microphthalmia

**Fig. 3.**
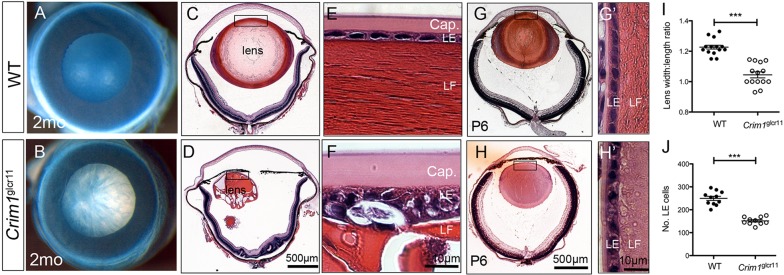
**Capsular rupture and LE-LE and LE-LF cell defects in *Crim1*^glcr11^ mutants.** (A,B) Absence of cataract in 2-month-old wild-type mouse lens (A), and dense cataract in age-matched *Crim1*^glcr11^ mutant mouse lens (B). (C,D) Histology of wild-type lens at 2 months shows no ocular abnormality (C), whereas there is severe cataract with posterior lens rupture in the *Crim1*^glcr11^ mutant (D). (E,F) Higher magnification of LE cells shows a thickened anterior capsule (Cap.) and vacuolization and detachment of LE cells from LF cells in *Crim1*^glcr11^ mutants (F), as compared with wild type (E). (G-H′) The morphological phenotype appears as early as P6 as the mutant develops a smaller lens with vacuolization at LE-LF cell junctions (H′), as compared with wild type (G′). (I,J) Quantification of lens width versus length ratio (I) and LE cell number (J) reveals altered morphology and decreased LE cell number in the mutant lens. *n*=5; ***P<0.001 (Student's *t*-test). Images shown are representative of four independent experiments.

Within 8 weeks of birth, all *Crim1*^glcr11^ mutants develop a posterior lens cataract and retinal dysplasia, and ∼19% of mutants exhibit microphthalmia (Fig. 3A,B, Fig. S2A,B). At later stages, the LE and LF cell compartments develop vacuoles that are frequently associated with lens capsular rupture and extrusion of the LF into either the anterior chamber or the vitreous (Fig. 3C-F). These gross structural defects can be detected as early as postnatal day (P) 6. In wild-type lenses, LE cells show an orderly alignment (Fig. 3G′), whereas in mutant lenses the LE cells develop vacuoles and exhibit a disrupted cellular architecture (Fig. 3H′). The lenses in mutant mice are also smaller, with an altered shape and fewer LE cells per section (152±6 cells) compared with those of wild-type controls (250±9 cells; Fig. 3G-J). In summary, *Crim1*^glcr11^ mutants develop multiple lens defects, suggesting that Crim1 mediates multiple cellular events during lens development.

### *Crim1*^glcr11^ mutants exhibit defective LE-LF cell adhesion, and LE cell polarity and proliferation

**Fig. 4.**
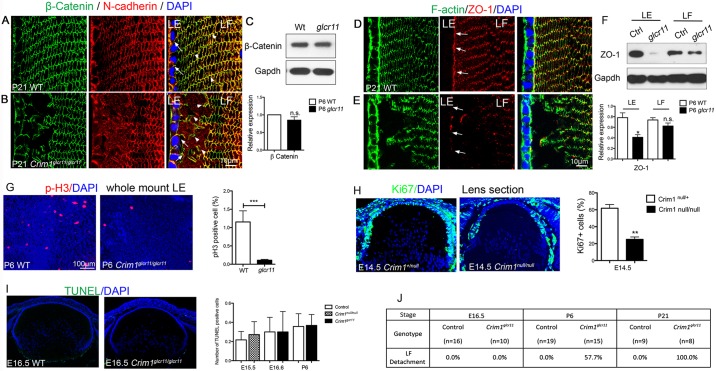
***Crim1*^glcr11^ defects in lens cell adhesion, polarity and proliferation.** (A-F) Altered expression patterns of adhesion proteins β-catenin and N-cadherin and the polarity protein ZO-1 in P21 *Crim1*^glcr11^ mutant LE cells (B,E, arrows point at LE-LF adhesion, arrowheads point at LF-LF adhesions) compared with wild type (A,D). Western blot shows that β-catenin levels are unchanged (C), whereas ZO-1 levels are significantly decreased in LE cells but not in LF cells (F). *n*=4. (G) Whole-mount immunostaining of phospho-Histone H3 reveals decreased proliferation in *Crim1*^glcr11^ LE cells. Quantitative analysis of the percentage of LE cells undergoing cell proliferation at P6 (right). Data are mean±s.d. for six independent experiments. (H) Immunostaining of Ki67 indicates decreased proliferation in *Crim1*^null^ LE cells. Quantitative analysis of the percentage of LE cells undergoing cell proliferation at E14.5 (right). *n*=3. (I) TUNEL assay. No TUNEL^+^ cells were found in the wild-type or *Crim1*^glcr11^ mutant lens. The average number of TUNEL^+^ cells per section is shown. *n*=3. (J) Quantification of LF detachment defects in E16.5, P6 and P21 *Crim1*^glcr11^ mutants. *P<0.05, **P<0.01, ***P<0.001; n.s., not significant (Student's *t*-test).

Cell adhesion, polarity, proliferation and apoptosis are important processes in lens development, and their perturbation can contribute to cataract formation. We first examined adhesion junctions by staining for N-cadherin and β-catenin in P21 lenses. In wild-type lenses, the adhesion junctions mainly reside at the cell-cell borders of LE cells, LE-LF junctions (Fig. 4A, arrows), and along the short ends of hexagonal LF cells (Fig. 4A, arrowheads). By contrast, the localization of N-cadherin and β-catenin in the anterior region of mutant lenses is severely disorganized (Fig. 4B, arrows, Fig. S3A). In particular, LE-LF adhesion is disrupted in *Crim1*^glcr11^ mutants (Fig. 4B, arrows), leading to the detachment of LF from LE cells (Fig. 4J). Altered cell adhesion is commonly coupled with defective apical-basal polarity. We therefore examined the expression of ZO-1 (Tjp1 – Mouse Genome Informatics), a tight junction marker, which revealed a loss of this polarity marker in LE cells (Fig. 4D-F, arrows, Fig. S3B,C). The decrease in ZO-1 expression is more severe in LE cells, suggesting that the LF cell phenotype might be secondary to detachment from LE cells.

**Fig. 5.**
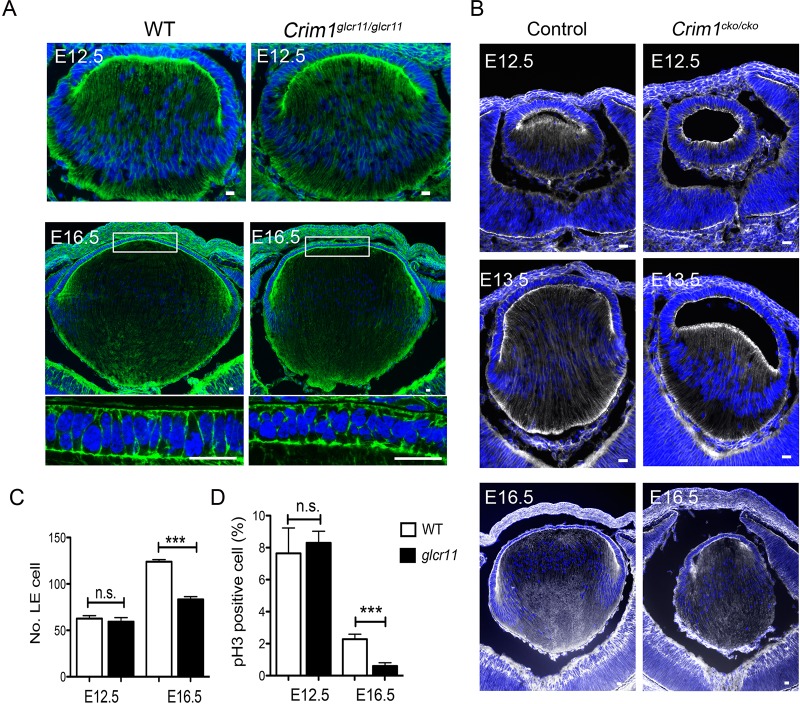
***Crim1*^glcr11^ and *Crim1*^cko^ mice exhibit lens development defects.** (A,B) F-actin staining with Phalloidin in *Crim1*^glcr11^ (A) and in *Crim1*^cko^ (B) lenses reveals a small lens of altered morphology. Images shown are representative of six independent experiments. (C,D) Quantification of total LE cell number (C) and the percentage of LE cells undergoing proliferation (D) at E12.5 and E16.5. *n*=4; ***P<0.001 (Student's *t*-test). Scale bars: 20 μm.

*Crim1*^glcr11^ mutants also develop smaller lenses with fewer LE cells (Fig. 3J). This phenotype could result from decreased proliferation or increased apoptosis. Whereas TUNEL assay and staining for active caspase 3 showed no difference between wild-type and mutant lenses (Fig. 4I; data not shown), examination of proliferating LE cells by immunofluorescent detection of phospho-histone H3 or Ki67 revealed decreased proliferation in mutant lenses (Fig. 4G,H). To determine whether Crim1 is required for early lens development, we examined lens morphology at various developmental stages. *Crim1*^glcr11^ mice start to exhibit disorganized LE cells as early as E16.5, and quantification of total LE cell number and phospho-histone H3^+^ cells showed a significant decrease in LE cells and in proliferating LE cells in E16.5 mutant lenses (Fig. 5A,C,D). Thus, Crim1 is required for normal LE cell adhesion, polarity and proliferation.

Crim1 is also important for the normal development of other tissues, such as the vascular system surrounding the lens and retina (Fan et al., 2014). To exclude the possibility that the lens defects are secondary to other ocular abnormalities, we obtained a *Crim1* conditional knockout (*Crim1*^flox^) allele (Fan et al., 2014) and crossed it with *Le-Cre*, which provides lens-specific Cre expression (Ashery-Padan et al., 2000). Similar to *Crim1*^glcr11^ and *Crim1*^null^, *Crim1*^flox/flox^*;Le-Cre* mouse mutants (henceforth referred to as *Crim1*^cko^) developed small eyes with altered lens morphology, but with earlier onset than *Crim1*^glcr11^ mutants, beginning at E12.5 (Fig. 5B). Thus, both *Crim1*^cko^ and *Crim1*^null^ mutants exhibit lens defects that resemble, but occur earlier than, those in *Crim1*^glcr11^ mutants. Apart from those affecting the eye, *Crim1*^glcr11^ mutants did not exhibit other discernible defects, suggesting that the developing eye is highly sensitive to *Crim1* loss-of-function. These data further suggest that *Crim1*^glcr11^ is a hypomorphic allele, and that the full-length transmembrane form of Crim1 is required for normal lens development.

### Crim1 colocalizes with β1 integrin in LE cell membranes

In *Xenopus* neuroepithelial cells, the Crim1 C-terminal cytoplasmic domain stabilizes the interaction between N-cadherin and β-catenin (Ponferrada et al., 2012). However, in *Crim1*^glcr11^ mutants, β-catenin colocalizes properly with N-cadherin, and β-catenin protein levels remain unchanged (Fig. 4B,C, Fig. S3A). This suggests that Crim1 acts through a different mechanism to regulate cell adhesion in lens tissue. Although Crim1 in cell lines plays a role in sequestering BMP and may act as a BMP antagonist (Wilkinson et al., 2003), we found that phospho-Smad 1/5/8, a downstream readout of BMP signaling, is unchanged in LE and LF cells in *Crim1*^glcr11^ mutants (Fig. S4A,B). Moreover, *Crim1*^null^ mice do not exhibit defects in lens induction or in early body axis patterning (data not shown), which are known BMP-dependent processes.

The morphology of *Crim1*^glcr11^ mutant LE cells is reminiscent of the disorganized lens epithelium associated with pathological epithelial-mesenchymal transition (EMT) in other mouse cataract mutants (Lovicu et al., 2002). Pathological lens EMT is associated with increased expression of mesenchymal α-smooth muscle actin, increased deposition of the ECM proteins collagen IV and fibronectin, and decreased expression of epithelial E-cadherin (Walker and Menko, 2009). However, we observed no change in E-cadherin, collagen IV or α-smooth muscle actin expression in *Crim1* mutants, effectively excluding the hypothesis that *Crim1* regulates EMT during lens development (Fig. S5). Furthermore, in *Crim1*^glcr11^ mutants neither LE cells nor LF cells showed alterations in phospho-Smad2 levels, a readout of TGFβ signaling and a recognized EMT modulator (Lovicu et al., 2002). Therefore, neither *Crim1* loss-of-function nor the truncated Crim1^glcr11^ protein causes pathological lens EMT.

**Fig. 6.**
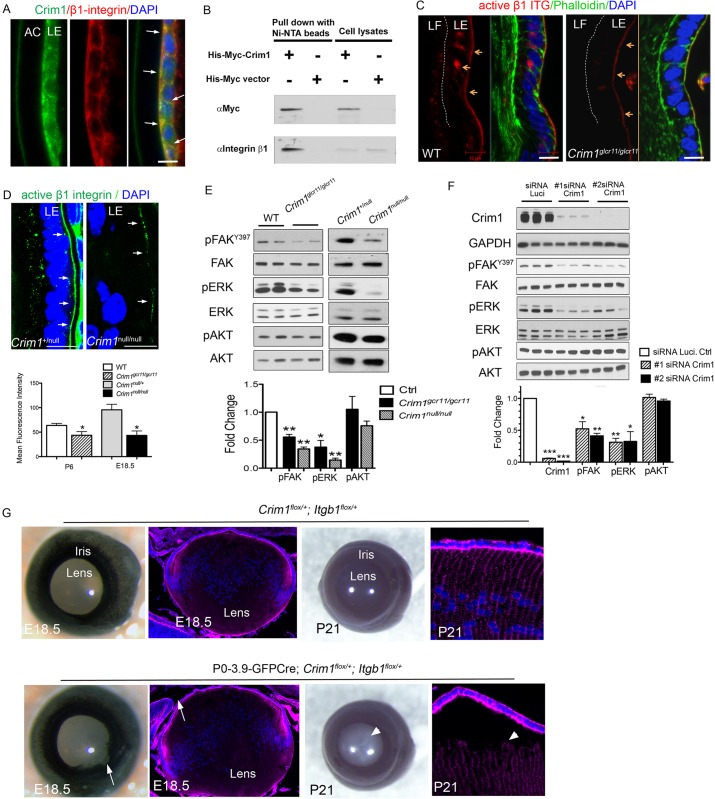
**Crim1 regulates integrin and FAK and ERK phosphorylation in lens development.** (A) Colocalization of β1 integrin and Crim1 (arrows) at LE cell-cell adhesions and at the basal surface of LE cells. (B) Co-immunoprecipitation of β1 integrin with His-Myc-tagged Crim1-FL in HEK 293T cells. (C,D) Immunostaining of active (9EG7) β1 integrin shows decreased staining in *Crim1*^glcr11^ (C) and *Crim1*^null^ (D) lenses. Arrows point to the LE cell basement membrane. Phalloidin stains the actin cytoskeleton; DAPI stains nuclei. Quantification of fluorescence intensity is shown beneath. *n*=3. (E) Western blot analysis of P6 wild-type and *Crim1*^glcr11^ lenses (left) and E18.5 control and *Crim1*^null^ lenses (right) with the indicated antibodies. *n*=5. (F) 21EM15 cells were treated with either of two siRNAs directed against *Crim1* for 48 h and then cell lysates were western blotted with the indicated antibodies. Each bar represents the mean of triplicates. (G) Compound heterozygous *P0-3.9-GFPCre;Crim1*^flox^^/+^*;Itgb1*^flox/+^ mice exhibit iris coloboma (arrows) and abnormal lens morphology at E18.5, and later develop bilateral cataract at P21 (arrowheads). *n*=6; all six P21 compound heterozygotes obtained were affected, as compared with none of four littermate controls. Arrowhead in P21 section indicates LE cell detachment from LF cells. Red, Phalloidin-stained actin cytoskeleton. Blue, DAPI-stained nuclei. *P<0.05, **P<0.01, ***P<0.001 (Student's *t*-test). Scale bars: 10 μm.

A conditional null mutation of *Itgb1* in the mouse lens shows defects in LE cell adhesion and loss of LE integrity that partly resemble those in the *Crim1* mutant lens (Samuelsson et al., 2007; Simirskii et al., 2007). Because β1 integrin is expressed in LE cell membranes and in LF cells (Bassnett et al., 1999; Samuelsson et al., 2007; Simirskii et al., 2007), we hypothesized that Crim1 might interact with β1 integrin, directly or indirectly, to regulate cell-cell adhesion in the developing lens. Indeed, we found that Crim1 bears an extracellular Arg-Gly-Asp (RGD) sequence, which is a well-known integrin-binding motif. Immunostaining of Crim1 and β1 integrin showed strong endogenous coexpression at the basal surface and cell-cell border of LE cells (Fig. 6A, arrows). Using co-immunoprecipitation, we also found that Crim1 can physically interact with β1 integrin (Fig. 6B). Immunostaining with an antibody against active β1 integrin showed significantly reduced levels in the *Crim1*^glcr11^ and *Crim1*^null^ lens equatorial zone (Fig. 6C,D).

To test whether *Itgb1* and *Crim1* function within the same genetic pathway *in vivo*, we performed a genetic interaction experiment by crossing *P0-3.9-GFPCre;Crim1*^flox/flox^ and *Itgb1*^flox/flox^. At E18.5, one compound heterozygous *P0-3.9-GFPCre;Crim1*^flox^^/+^*;Itgb1*^flox/+^ mouse out of four displayed iris coloboma similar to that seen in human *CRIM1* haploinsufficiency (Fig. 6G). The phenotype became more severe as six out of six P21 compound heterozygotes exhibited bilateral cataract (Fig. 6G, arrowhead), compared with none of four littermate controls. Immunostaining of compound heterozygote lenses showed LE cell detachment from LF cells, similar to that seen in *Crim1* mutants. This genetic interaction is consistent with the idea that Crim1 interacts with the β1 integrin signaling pathway, and that it partly regulates lens morphogenesis by this mechanism.

### Crim1 regulates FAK and ERK phosphorylation

An early event in integrin activation is the phosphorylation of FAK (Ptk2), which drives the actin cytoskeletal reorganization required for cell spreading, migration and polarity. Subsequent events can include activation of ERK and AKT, which regulate cell proliferation and survival, respectively (Legate et al., 2009). Western blot analyses of *Crim1*^glcr11^ and *Crim1*^null^ lenses showed statistically significant decreases in phospho-FAK (pFAK) to 56±10% and 34±6% of wild-type levels and in phospho-ERK (pERK) to 37±13% and 14±7% of wild-type levels, respectively (Fig. 6E; see legend for *P*-values). We also examined whether reductions in Crim1 regulated AKT phosphorylation. However, no significant change in phospho-AKT (pAKT) was observed (Fig. 6E). Lastly, to confirm that Crim1 regulates integrin signaling, we performed Crim1 knockdowns in 21EM15 LE cells (Terrell et al., 2015). When endogenous Crim1 was knocked down to <10% of wild-type levels by either of two siRNAs, pFAK and pERK, but not pAKT, were downregulated in a statistically significant fashion to ∼50% and ∼35% of control levels, respectively (Fig. 6F). Crim1 thus appears to play a role in regulating the phosphorylation status of FAK and ERK, and hence FAK and ERK signaling in lens morphogenesis.

### Membrane-bound Crim1 is crucial to activate β1 integrin signaling

**Fig. 7.**
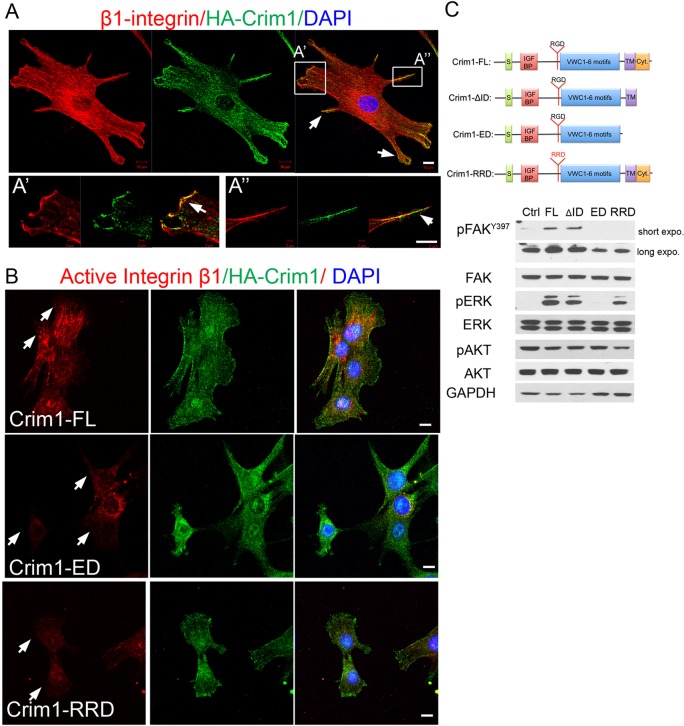
**Crim1 colocalizes with integrin and activates the integrin-FAK-ERK signaling pathway.** (A) Crim1 and β1 integrin colocalize at the tip of lamellipodia (A′, arrows) and filopodia (A″, arrowhead) in 21EM15 cells. (B) Immunocytochemical detection of active (9EG7) β1 integrin on the cell surface of 21EM15 lens cells transfected with HA-tagged Crim1-FL vector (top row), Crim1-ED (middle row) or Crim1-RRD (bottom row). Arrows point to active integrin staining. (C) Expression constructs representing Myc-tagged full-length Crim1 (FL), Crim1 extracellular domain only (ED), Crim1 lacking the intracellular domain (ΔID), and Crim1 carrying RGD in place of RRD (Crim1-RRD) were transfected into HEK 293T cells for 48 h and then western blotted with the indicated antibodies. pFAK^Y397^ and pERK are induced by FL and ΔID, whereas RRD induces pERK but not pFAK^Y397^. Ctrl is vector only. Images shown are representative of three independent experiments. Scale bars: 10 μm.

To determine the subcellular localization of Crim1, we prepared an HA-tagged Crim1 construct and transfected it into 21EM15 cells. HA-tagged Crim1 colocalized at the tips of lamellipodia and filopodia with β1 integrin (Fig. 7A′,A″). Next, to determine which Crim1 domains are required for FAK and ERK phosphorylation, we examined FAK and ERK activity in 21EM15 cells that overexpressed one of four constructs: (1) full-length Crim1 (Crim1-FL); (2) Crim1 without the intracellular domain (Crim1-ΔID); (3) Crim1 with only the extracellular domain (Crim1-ED); or (4) Crim1 with a G315R mutation in its RGD motif (Crim1-RRD).

Cells overexpressing HA-tagged Crim1-FL showed an increase in active β1 integrin activity compared with cells overexpressing Crim1-ED or Crim1-RRD (Fig. 7B, arrows). Similarly, overexpression of Crim1-FL or Crim1-ΔID effectively induced FAK and ERK phosphorylation, whereas Crim1-ED overexpression failed to activate FAK or ERK (Fig. 7C). These results indicate that membrane-bound Crim1, but not the cytoplasmic tail, is required for FAK phosphorylation. Notably, overexpression of the Crim1-RRD mutant also failed to upregulate pFAK, but it did upregulate pERK, indicating that the Crim1 RGD motif is required for FAK phosphorylation and for discriminating between these two signaling pathways. Together with results from the knockdown and *in vivo* experiments, these data demonstrate that Crim1 can regulate integrin activity and FAK and ERK signaling in lens development.

## DISCUSSION

### Roles for *Crim1* in lens development

We identified an ENU-induced mouse mutation that truncates the Crim1 protein prior to its transmembrane domain, thus creating a novel allele, *Crim1*^g^^lcr11^, with which to study Crim1 function. The *Crim1*^g^^lcr11^ allele is deficient for full-length membrane-bound Crim1, but expresses a truncated extracellular form that resembles a normal Crim1 proteolytic product. Several lines of evidence suggest that the truncated form expressed by the mutant, and by inference the Crim1 proteolysis product that it resembles, might not have a major role in lens morphogenesis. First, in a complementation test, the *Crim1*^g^^lcr11^ allele behaves as a classic hypomorphic loss-of-function allele. Second, unlike full-length Crim1, Crim1-ED overexpression is unable to activate FAK or ERK, suggesting that the truncated extracellular form encoded by the *Crim1*^g^^lcr11^ allele would also lack this function. Lastly, the proteolytic product only represents a small fraction of the total Crim1 gene product in the wild-type lens, as revealed by western blot. Using the hypomorphic *Crim1*^g^^lcr11^ allele, along with the existing *Crim1*^null^ and *Crim1*^cko^ alleles, we uncovered a novel function for Crim1 in regulating LE cell behavior *in vivo* and a mechanistic link between Crim1 and β1 integrin-dependent regulation of lens morphogenesis.

Recently, *CRIM1* haploinsufficiency has been linked to human MACOM syndrome, an ocular disease characterized by microcornea, iris coloboma and increased axial length with severe myopia (Beleggia et al., 2015). Affected individuals in this family carry a 22 kb heterozygous deletion that encompasses terminal exons 14-17 of *CRIM1* as well as most of the 3′-UTR of an adjacent gene, *FEZ2.* The human MACOM phenotype might reflect the effect of *CRIM1* loss-of-function or, since *Fez2* is expressed in mouse E11.5 and E12.5 lens according to the ocular lens gene discovery tool *iSyTE* (Lachke et al., 2012) and potentially in other ocular tissues, the combined effects of *CRIM1* and *FEZ2* loss-of-function. The latter scenario could explain why the MACOM phenotype involves macrophthalmia without cataracts, whereas all three mouse *Crim1* loss-of-function models exhibit microphthalmia and congenital cataracts. Alternatively, the difference in the mouse and human phenotypes might reflect differences in ocular development, in modifying alleles, or in Crim1 expression in the two species. Yet another explanation for the discrepancy in phenotypes is simply that heterozygosity for human *CRIM1* does not cause sufficient loss-of-function to produce a cataract phenotype.

Although the role of *CRIM1* in human ocular pathology remains open, our work provides definitive *in vivo* evidence that *Crim1* is required in lens development to maintain LE cell polarity, adhesion and proliferation. The membrane localization of Crim1 protein is essential for these events, as loss of Crim1 membrane localization impairs LE cell shape and cell adhesion. Indeed, Crim1 specifically localizes to the leading edge of cell protrusions, where actin cytoskeleton remodeling is highly active during the acquisition of polarity by LE cells.

### A link between Crim1 and β1 integrin signaling in lens development

Several lines of evidence suggest that Crim1 function during lens development involves the regulation of β1 integrin signaling. First, Crim1 and β1 integrin are coexpressed in LE cells and at LE-LE cell interfaces, and the level of activated β1 integrin in LE cells depends upon *Crim1* function. Crim1 and β1 integrin also possess the potential to physically interact with each other, as shown by co-immunoprecipitation. In addition, in cells transfected with Crim1, Crim1 and β1 integrin colocalize to the tips of lamellipodia and filopodia. Thus, Crim1 and β1 integrin appear to reside in close physical proximity in LE cells. Since the interaction of integrins expressed at the LE cell basal surface with ECM components of the lens capsule basement membrane is well supported (Wederell and de longh, 2006), the interaction between integrins and Crim1 at the lateral surfaces of LE cells could provide a similar mechanism for cell adhesion between LE cells.

A second line of evidence that supports an interaction between Crim1 and β1 integrin signaling is that Crim1 function, and specifically that of membrane-bound, RGD-containing Crim1, is required for the phosphorylation of FAK, a key proximal mediator of integrin signaling. The effect of mutation in the Crim1 RGD motif in abrogating FAK phosphorylation supports this mechanism. The specific localization of Crim1 to LE cell membranes, coupled with the role of Crim1 in the regulation of integrin signaling, could provide a mechanism to restrict the activation of integrin in LE cells.

A last line of evidence that Crim1 function intersects with integrin signaling is genetic. The lens phenotypes associated with *Itgb1* and with integrin-linked kinase (*Ilk*) mutants bear some similarity to the *Crim1* mutant lens phenotype. In a conditional *Itgb1* knockout, LE cells exhibit vacuolization with increased apoptosis, while mice deficient for *Ilk* exhibit LE cell disorganization, decreased LE cell numbers, decreased cell polarity with disturbed ZO-1 expression, and decreased pERK activity in LE cells (Cammas et al., 2012). These phenotypic similarities could be consistent with a role for Crim1 in modulating β1 integrin signaling during lens development. To test this hypothesis genetically, we intercrossed the *Crim1*^null^ and *Itgb1*^flox^ alleles, the latter conditionally inactivated via *P0-3.9-GFPCre*, and examined the resulting compound heterozygotes for evidence of synthetic phenotypic enhancement. Interestingly, *Crim1*^null^ and *Itgb1*^flox^ compound heterozygotes displayed iris coloboma and cataract, confirming that *Crim1* and *Itgb1* genetically interact.

In summary, Crim1 appears to function as an adhesion protein, is highly enriched in developing lens, and its perturbation in mouse causes cataract with altered LE cell adhesion, polarity and proliferation. In addition, Crim1 appears to influence the integrin signaling that is crucial for lens development. Crim1 deficiency disturbs this regulation and leads to cataract. Interestingly, Crim1 is also expressed in the developing kidney, and *Crim1* deficiency leads to a dilated and disorganized renal capillary network (Wilkinson et al., 2007). This phenotype resembles that in *Itga3;Itgb1* knockout mice (Kreidberg et al., 1996), suggesting that Crim1 might regulate integrin signaling in other developing organs in addition to the eye. It will be interesting to investigate whether the function of Crim1 uncovered here is also deployed in other tissues during development.

## MATERIALS AND METHODS

### Mouse strains

*Crim1*^glcr11^ mice were identified as part of a phenotype-driven screen to detect mutagenized mice with glaucoma-relevant and cataractous phenotypes. The *Crim1*^glcr11^ mutation was induced with N-ethyl-N-nitrosourea (ENU) on a C57BL/6J genetic background and outcrossed to C3A.BLiA-Pde6b+/J for more than three generations. *Crim1*^glcr11^ was initially mapped to a 26 Mb region between Massachusetts Institute of Technology (MIT) SSLP markers D17mit20 and D17mit243, with fine mapping to an 8 Mb region flanked by D17mit160 and D17mit187. *Crim1*^flox^ and *Crim1*^null^ alleles were obtained from Dr Richard Lang [Cincinnati Children's Hospital (Fan et al., 2014)]. *Crim1*^cko^ allele was generated by crossing the *Crim1*^flox/flox^ mice with *Le-Cre* mice, which express Cre recombinase specifically in lens tissue (Ashery-Padan et al., 2000). The *Itgb1*^flox^ allele was purchased from the Jackson Lab. *P0-3.9-GFPCre* mice, which express an EGFP-Cre recombinase fusion protein under the control of the *Pax6* lens ectoderm enhancer and the *Pax6 P0* promoter, were maintained in a FVB/N background (Rowan et al., 2008). All mouse experiments reported here were carried out under HMS IACUC protocol no. 750.

Genotyping was performed using OneTaq polymerase (NEB) and the following primers (5′-3′, forward and reverse): *Crim1*^glcr11^, AGTCACCCTGGCACATCATT and ATGTCCGTACCGAACCAGTC (the 297 bp PCR product was then purified for sequencing using the forward primer; *Crim1*^Glcr11^ carries a G433A mutation in intron 13 of the *Crim1* gene); *Crim1*^flox^, TCTGGATCAGCAGAGTCAATTAGATGC and CTCCACACGAGTTTCAATGAGCTGAGC (PCR products of 328 bp for wild type and 1.2 kb for mutant); *Le-Cre*, ACACCAGAGACGGAAATCCATC and GGCCAGCTAAACATGCTTCA (PCR product 500 bp); *Crim1*^null^, TCTGGATCAGCAGAGTCAATTAGATGC and GTGGTGATGACTTGGCTAGTCCAATGG (PCR products of 3.5 kb for wild type and 1.7 kb for mutant); *P0-3.9-GFPCre*, ACACCAGAGACGGAAATCCATC and GGCCAGCTAAACATGCTTCA.

### Whole-genome sequencing (WGS)

To localize the causative mutation, we used WGS methods. Briefly, the genomic DNA of *Crim1*^glcr11/glcr11^ mutant spleen tissue was isolated using the DNeasy Blood & Tissue Kit (Qiagen). Library preparation was performed using the AIR Genomic DNA Sequencing Kit (Illumina) according to the manufacturer's protocol. The prepared sample was then sequenced using the Illumina HiSeq 2000 system. Novoalign (Novocraft Technologies) and SAMTools were used to map the sequence reads to the mouse reference genome mm9. Default settings were used for all options. Potential PCR duplicates were removed using Picard software (http://broadinstitute.github.io/picard/). Both single nucleotide variants (SNVs) and small insertions and deletions (indels) were called using the Genomic Analysis Toolkit (GATK) pipeline (McKenna et al., 2010). Known variants present in dbSNP132 and Mouse Genomes Project (Keane et al., 2011) were filtered out. All identified variants were annotated using the ANNOVAR software (http://annovar.openbioinformatics.org/). Based on genetic mapping experiments, we localized the mutation to chr17:57,584,601-83,619,328. We identified mutations that are shared by two *Crim1*^glcr11^ mutant mice within this 26 Mb region.

### Hematoxylin and Eosin (H&E) staining

Enucleated eyes were fixed in 4% formaldehyde overnight at 4°C and processed for paraffin embedding. Serial sagittal sections passing through the optic nerve were collected, stained with H&E and analyzed for pathological alterations.

### Immunohistochemistry

Mouse lenses were fixed in 4% formaldehyde for 30 min at room temperature, incubated in 10% sucrose overnight at 4°C, embedded in OCT compound (Tissue-Tek), and cryosectioned at 10 μm. Frozen sections were incubated in 0.3% Triton X-100 for 30 min and blocked in 5% chicken serum for 1 h. Primary antibody was added and incubated overnight at 4°C. Chick secondary antibodies (Invitrogen) were incubated at room temperature for 1 h. Antibodies used were: Crim1 (#ab2249, 1:100, Millipore), N-cadherin (#MNCD2-A2, 1:50, Developmental Studies Hybridoma Bank), β-catenin (#610153, 1:400, BD Biosciences), E-cadherin (#610181, 1:100, BD Transduction), ZO-1 (#33-9100, 1:200, Invitrogen), phospho-Histone H3 (#9701, 1:400, Cell Signaling), Ki67 (#9129, 1:400, Cell Signaling), pSmad1/5/8 (#9511, 1:1000, Cell Signaling), pFAK^Y397^ (#8556, 1:1000, Cell Signaling), FAK (#3285, 1:1000, Cell Signaling), pERK (#4370, 1:1000, Cell Signaling), ERK (#9102, 1:1000, Cell Signaling), pAKT^S473^ (#4060, 1:1000, Cell Signaling), AKT (#4691, 1:1000, Cell Signaling), Smad4 (#9515, 1:1000, Cell Signaling), Gapdh (#2118, 1:1000, Cell Signaling), β1 integrin (MAB1997, 1:200, Millipore), β1 integrin (#4706, 1:200, Cell Signaling), active β1 integrin (#550531, 1:25, BD Biosciences), Prox1 (ab38692, 1:200, Abcam), αSma (clone 1A4, 1:50, DAKO), anti-Myc (9E10, 1:200, Sigma) and anti-HA tag (ab18181, 1:200, Abcam). Alexa Fluor 488 Phalloidin was from Invitrogen (A12379, 1:200). Images were acquired with a Zeiss LSM780 inverted confocal microscope.

### TUNEL staining

TUNEL staining was performed according to the manufacturer's protocol (#17-141, Millipore).

### Western blots

Eyes were enucleated from control and *Crim1*^g^^lcr11^ mice. Retinas were removed and processed separately. Separating lens epithelium and lens fiber was performed as described (Sugiyama and McAvoy, 2012). In brief, lenses were dissected from eyeballs. A small tear was made on the posterior capsule. Capsule was then peered off along the tears and lens fiber mass was collected. The remaining capsule containing lens epithelium was collected for further analysis. Tissue was homogenized in RIPA lysis buffer (Sigma) with protease inhibitor cocktail (Roche, 04693124001) and centrifuged at 16,000 ***g*** for 12 min. The cellular lysate was resolved by 4-20% gradient SDS PAGE. Proteins were blotted to a PVDF membrane and incubated overnight at 4°C with primary antibody then incubated with HRP-conjugated secondary antibody (Jackson ImmunoResearch). Protein was visualized using chemiluminescent substrate (Pierce Biotechnology).

### Immunoprecipitation

HEK 293T cells were transfected at 60% confluency using calcium phosphate transfection. To detect Crim1 and β1 integrin interaction, C-terminal His- and Myc-tagged mouse Crim1 was expressed in HEK 293T cells; the empty vector was used as a negative control. 48 h post-transfection, cells were lysed in lysis buffer (50 mM NaH_2_PO_4_, 300 mM NaCl, 10 mM imidazole, 0.5% Triton X-100, 1× protease inhibitor, pH 8.0). Cell lysates were centrifuged at 15,000 ***g*** for 10 min at 4°C; supernatants were incubated with Ni-NTA resin (Qiagen) at 4°C for 2 h to precipitate His-Myc-Crim1. The resin was washed three times with washing buffer (50 mM NaH_2_PO_4_, 300 mM NaCl, 20 mM imidazole, 0.5% Triton X-100, pH 8.0) and heated at 95°C for 10 min. The precipitates were resolved on 10% SDS-PAGE gels and blotted with mouse anti-Myc or rabbit anti-β1 integrin antibodies at 1:1000 dilution.

### Plasmids

His-Myc-Crim1-FL, His-Myc-Crim1-ED and His-Myc-Crim1-ΔID plasmids were a gift from Dr Richard Lang (Ponferrada et al., 2012). The His-Myc-Crim1-RRD construct was made using a site-directed mutagenesis kit (NEB), followed by sequencing to confirm the G943A mutation. HA-Flag-Crim1 plasmid was generated through Gateway cloning (Invitrogen). In brief, the entry clone containing *Crim1* cDNA was introduced to the pDEST-Flag-HA vector (a kind gift from Dr Karen Cichowski, Brigham and Women's Hospital, MA, USA).

### Cell culture

The mouse lens epithelial cell line 21EM15, a generous gift of Dr John Reddan (Oakland University, MI, USA) was cultured under standard conditions (DMEM, 10% fetal bovine serum, penicillin-streptomycin, at 37°C in a water saturated atmosphere with 5% CO_2_). Cells were transfected with siRNAs at 10 nM final concentrations for 48 h using RNAiMAX (Invitrogen). siRNA target sequences used were: #1 Crim1, 5′-GUUUGUGAGGUGGGAUCUA-3′; #2 Crim1, 5′-CUGCGUUUAUGGCUUCAAA-3′; and Luciferase control (Dharmacon).

To infect 21EM15 cells, lentivirus preparations containing target constructs were used according to the manufacturer's protocol (Clontech). In brief, a lentiviral supernatant was used at ∼5×10^5^ TU to infect 7.5×10^5^ 21EM15 cells in the presence of 8 μg/ml polybrene (Sigma) for 6 h at 37°C. Cell lines were selected at a final concentration of 4 μg/ml puromycin for 2 days.
